# Is the Patient Activation Measure a valid measure of osteoarthritis self-management attitudes and capabilities? Results of a Rasch analysis

**DOI:** 10.1186/s12955-020-01364-6

**Published:** 2020-05-05

**Authors:** J. P. Eyles, M. Ferreira, K. Mills, B. R. Lucas, S. R. Robbins, M. Williams, H. Lee, S. Appleton, D. J. Hunter

**Affiliations:** 1 Kolling Institute of Medical Research, Institute of Bone and Joint Research, Faculty of Medicine and Health, University of Sydney, Sydney, Australia; 2 Department of Rheumatology, Royal North Shore Hospital , Sydney, Australia; 3 Physiotherapy Department, Royal North Shore Hospital, Sydney, Australia; 4 Faculty of Medicine and Health Sciences, Macquarie University, Sydney, Australia; 5 Rehabilitation Department, Hunters Hill Private Hospital, Sydney, Australia; 6 Physiotherapy Department, Mount Wilga Private Hospital, Sydney, Australia

**Keywords:** Measurement properties, Rasch, Osteoarthritis, Psychometrics, Patient activation

## Abstract

**Background:**

The Patient Activation Measure (PAM-13) was developed using Rasch analysis to assess knowledge, skills and confidence in the management of one’s health. Previous studies report positive relationships between PAM-13 scores, self-management behaviours and longitudinal health outcomes in adults with chronic disease. There is little extant measurement property evidence for the use of PAM-13 in specific osteoarthritis (OA) populations. This study tested measurement properties of the PAM-13 in people living with hip and knee OA.

**Methods:**

Item response frequency analysis was conducted. Rasch analysis evaluated the fit of the PAM-13 data to the Rasch model. Model-data fit was evaluated using infit and outfit statistics; person/item reliability and person separation indices were computed. Unidimensionality was evaluated using Principal Components Analysis of Rasch residuals and the data were assessed for item redundancy. Differential Item Functioning (DIF) examined bias in respondent subgroups and correlations tested relationships between PAM-13 and other patient-reported outcomes.

**Results:**

Two-hundred-and-seventeen PAM-13 surveys were completed; there were no missing responses, floor or ceiling effects. Person and item reliability were acceptable (0.98 and 0.87 respectively) with good separation (person separation index 2.58). Unidimensionality was evaluated, with 49.4% of the variance explained by the first eigenvector. There was evidence of potential local response-dependence. The Rasch fit statistics were acceptable (except for item-2). There were some issues identified with targeting of the PAM-13 items to people with higher ability and the item difficulty order was different to that proposed in original cohorts. Significant DIF was identified for sex and educational level for a small number of items. PAM-13 scores were moderately correlated with depressive symptoms on the Depression Anxiety Stress Scale and Assessment of Quality of Life-6D. There were small correlations between PAM-13 and Knee injury and Osteoarthritis Outcome Score pain and activities of daily living scores.

**Conclusions:**

This study provides some evidence of adequate person and item reliability, unidimensionality, and construct validity to support the use of PAM-13 to measure patient activation in people living with hip and knee OA. Possible limitations regarding targeting, different item difficulty order, DIF and local response dependence should be investigated in future research.

## Background

Osteoarthritis (OA) is a prevalent, painful condition and a leading cause of global disability [1]. As a costly [2], chronic, incurable disease, self-management interventions are recommended for the management of osteoarthritis (OA) [3]. Two systematic reviews have evaluated the effects of self-management interventions that included OA patients. The first demonstrated evidence of small to moderate effects in terms of pain and functional improvements conferred by arthritis self-management interventions [4]. The second was concerned specifically with OA self-management education programs and found no or small benefits from these programs [5]. These reviews highlight that measures of pain and function are the most common primary outcomes for self-management interventions [4, 5]. Whilst pain and function are obviously important to this population, there is a disparity between the aims of self-management programs and the outcomes used to assess efficacy. A more meaningful measurement of program efficacy would be to measure OA self-management attitudes and capabilities [5], which have been recognised as comparatively neglected domains [6].

The measurement of OA self-management attitudes and capabilities requires validated instruments that have demonstrated adequate measurement properties in populations with OA [7]. Measurement properties refer to the ability of the instrument to accurately and comprehensively measure the specified construct [8] (e.g. internal consistency, reliability, validity). A recent systematic review of instruments assessing OA self-management attitudes and capabilities found that there was very little measurement property evidence available and that further research was needed to fill this knowledge gap [9].

An instrument identified in the review was the Patient Activation Measure (PAM-13); a patient-reported outcome assessing the knowledge, skill and confidence in the management of one’s health [10]. The measurement properties of the PAM-13 have been studied in populations with varying chronic conditions including mental illness [11], neurological disorders [12] and multimorbidity [13, 14]. Two previous studies investigated measurement properties of the PAM-13 in OA populations. The first translated the PAM-13 into Korean and provided some evidence of adequate internal consistency and structural validity [15]. The second examined the responsiveness of PAM-13 in a sample of people with “arthritis”, not specifically OA [16]. This study aims to provide further evidence of measurement properties of the PAM-13 in people living with OA.

Several large cohort studies report that higher levels of patient activation measured by the PAM-13 predict better self-management behaviours and longitudinal health outcomes in adults with chronic disease [17–19]. This considered, it may be possible to predict patient outcomes following OA management programs using PAM-13 scores. This would enable the identification of people likely to experience a positive treatment effect. These people could then be prioritised for participation in these programs. Conversely, people reporting poorer self-management attitudes and capabilities may be identified and targeted for supplementary therapies (e.g. motivational coaching). Further, the efficacy of OA management programs could be measured in terms of change in patient activation. Before these potential uses of the PAM-13 are tested, it is important to establish that its measurement properties are acceptable in the OA population.

The PAM-13 developers used Rasch analysis to construct the instrument according to the Rasch measurement model [10]. The Rasch model determines the measurement requirements for the construction of interval level measurement scales [20]. A major advantage of using instruments developed using Rasch analysis is that the measurements can be assumed to produce interval level variables, hence, statistical tests requiring interval level variables can be used to report the results of clinical studies [21]. Rasch analysis also provides a unified measurement approach to test the validity of an instrument developed using this method when it is tested in a different population of patients [7]. This study had the following aims:

i) To test the measurement properties including reliability (internal consistency), unidimensionality (structural validity) and construct validity and floor/ceiling effects of the PAM-13 in people with hip and knee OA.
ii) To examine the relationships between PAM-13 scores and psychological, quality of life and disease-specific outcomes.

## Methods

### Participants

This cohort study comprised participants of OA management programs (OAMP). Participants were recruited directly from Royal North Shore, Ryde (major teaching hospitals), Hunter’s Hill Private and Mount Wilga (private metropolitan hospitals) hospitals in Australia via referral from rheumatologists, orthopaedic surgeons and general practitioners or joint arthroplasty waiting lists. People with symptomatic and radiographic hip and knee OA were eligible if they reported pain in the affected knee/hip on most days of the past month. Details of the program are published elsewhere [22]. Ethical approval for this study in accordance with the Declaration of Helsinki was provided by Human Research Ethics Committees: NSPHEC 2016-LNR-007; NSPHEC 2017-LNR-005 and LNRl16/HAWKE/14. Participants provided written consent to take part in this study prior to the start of the investigation.

### Data

All data were collected at the baseline assessment of OAMP as part of the normal clinical pathway. Signal joint, the predominant site of OA, was determined by clinical and radiographic examination. Anthropometric measurements were undertaken using a standardised protocol [23]. Participants rated their average pain on the day of assessment using a Numeric Rating Scale (0 indicated no pain and 10 the most pain imaginable) [24]. Patient-reported outcomes were collected electronically as described below.

#### Patient Activation Measure-13

Participants rated their level of agreement with 13 statements (Table 1) using a 4-point Likert scale: Totally Disagree, Disagree, Agree, Totally Agree and Not Applicable (N/A). This outcome assumes that Item-1 is the easiest to endorse, and each subsequent item is more difficult to endorse than the one before [10]. The response (range 1–4) to the items are added to calculate a raw score. Responses of “not applicable” (N/A) are treated as missing. Scoring of the PAM-13 allows for any number of missing values, both items that are left blank and those with “not applicable” responses [25]. A continuous activation score is computed from the raw score using an empirically derived calibration table by Insignia Health (after January 2014). Total scores range from 0 (no activation) to 100 (high activation) [10]. PAM-13 score thresholds are used to assign four stages of activation in order of ascending activation: 1. “Believes active role is important”; 2. “Confidence and knowledge to take action”; 3. “Taking action”; 4. “Staying the course under stress” [10].

**Table 1:** Patient Activation Measure- 13 items and mean scores of responses

PAM-13 items	N	Mean^a^ (SD)
1. When all is said and done, I am the person who is responsible for taking care of my health	217	3.4 (0.73)
2. Taking an active role in my own health care is the most important thing that affects my health	217	3.4 (0.77)
3. I am confident I can help prevent or reduce problems associated with my health	217	3.3 (0.77)
4. I know what each of my prescribed medications do	200	3.3 (0.70)
5. I am confident that I can tell whether I need to go to the doctor or whether I can take care of a health problem myself	215	3.2 (0.70)
6. I am confident that I can tell a doctor concerns I have even when he or she does not ask	217	3.3 (0.65)
7. I am confident that I can follow through on medical treatments I may need to do at home	216	3.2 (0.67)
8. I understand my health problems and what causes them	215	3.1 (0.71)
9. I know what treatments are available for my health problems	212	2.8 (0.75)
10. I have been able to maintain (keep up with) lifestyle changes, like eating right or exercising	210	2.8 (0.70)
11. I know how to prevent problems with my health	208	2.6 (0.70)
12. I am confident I can figure out solutions when new problems arise with my health	212	2.8 (0.70)
13. I am confident that I can maintain lifestyle changes, like eating right and exercising, even during times of stress	216	2.8 (0.70)

^a^PAM items are scored using “Totally Disagree” = 1, “Disagree” = 2, “Agree” = 3, “Totally Agree” = 4

#### The hip disability and osteoarthritis outcome score (HOOS) and knee injury and osteoarthritis outcome score (KOOS)

The HOOS [26] and KOOS [27] are disease-specific measures that have been validated in people with OA. Participants rate their symptoms, stiffness, pain, physical function, recreational activities and quality of life on a 5-point Likert scale (0–4). The responses for the six subscales are summed and transformed to comprise six independent subscores; lower scores indicate worse problems.

#### The depression, anxiety and stress scale (DASS-21)

Participants rate their level of agreement with 21 statements using a 4-point Likert scale (0–3). The DASS-21 subscores indicate the presence/absence of symptoms of depression, anxiety and stress [28]. Higher scores indicate worse symptoms.

#### Assessment of quality of life (AQoL-6D)

Participants respond to questions or statements rated using four, five, or six point scales. Six dimensions are reported separately including independent living, relationships, mental health, coping, pain and senses which are combined for a standardised AQoL index. Higher scores indicate a worse quality of life [29].

### Statistical analysis

Descriptive statistics and correlations were processed using SPSS (Version 22.0, Armonk NY: IBM Corp, USA) software. The PAM-13 responses were compared to the Rasch model with Rasch analysis [20] using Winsteps (version 4.0.1 Linacre, J. M. (2017) Winsteps® Rasch measurement computer program. Beaverton, Oregon: Winsteps.com).

#### Item response frequency analysis

Item response analysis was conducted to demonstrate data quality [30]. The frequencies of each response option and missing responses were reported for each item. Floor and ceiling effects were confirmed if ≥15% of respondents answered “totally disagree” or “totally agree” to all items respectively [31].

#### Rasch model overview

The PAM-13 was originally developed using Rasch analysis [10]. A Rasch analysis compares individual items or responses of a patient-reported outcome measure with a Rasch model (RM) [21]. Comparison to a Rasch Model provides insight into whether scores obtained for individual items of the outcome measure can be added together to create an overall score. More specifically, it assists in determining whether the outcome measure possesses the properties of an interval scale or whether each item is stand-alone.

The RM assumes that responses to the items of an outcome scale are affected by the ability of the person and the difficulty of the item [32]. In Rasch analysis, metrics are calculated to determine whether the relationships between the ability of the person and the difficulty of the item in the study data are consistent with what would be expected to fit the RM and that the assumptions of the RM are met. In outcome scales that use ordered response categories, such as Likert scales used in the PAM-13, the partial credit Rasch model (PCM) can be used. The PCM allows for differing levels of response, between complete agreement and complete disagreement, with each item on the scale. Therefore, each item can be partially agreed, or disagreed with by a respondent. ‘Person ability’ is calculated using the number of items of the instrument that a person agreed or partially agreed with. ‘Item difficulty’ is estimated using the number of persons in the sample who agreed or partially agreed with an item [32]. The relationship between person ability and item difficulty is clearly depicted on person-item maps. Measures of fit are used to assess whether the instrument conforms to RM requirements; infit and outfit statistics are used to indicate how accurately or predictably data fit the model [33]. There is not complete agreement about the influence of sample size on fit statistics, however, a sample of 200 participants has been recommended [34]. For this study, we aimed to recruit 250 to account for 20% non-completion rate.

#### Reliability and separation

In Rasch analysis, the *person reliability index* estimates the probability that the ordering of persons (based on their abilities) is preserved when they respond to further items measuring the same construct. The *Item reliability index* indicates the probability that the order of the items (based on difficulty) would be the same if the same construct was measured in a similar but independent sample of people [32]. The *person separation index* tests if the instrument is sensitive enough to distinguish between people with high and low abilities. Thresholds for acceptable indices were set at > 0.8 for item reliability, > 0.8 for person reliability and > 2 for person separation index [31, 33]. The person-item map was used as a pictorial representation of how well the difficulty of the items aligned with the abilities of the persons who completed the survey. The alignment between item difficulty and person ability is referred to as ‘targeting’ [32].

#### Rasch model fit analysis

The partial credit model was used to examine model-data fit; it was chosen because the PAM-13 items were measured on a four-point Likert scale with ordinal response options [32]. Point-measure correlations were estimated to determine whether item responses aligned with person abilities. Point measure correlations > 0.5 were considered acceptable. Infit and outfit statistics (expressed in mean square (MnSq)) indicated how well the data fit the RM. Values between 0.5 and 1.5 MnSq were considered acceptable [32]. An approximate global log-likelihood chi-squared statistic for overall goodness of fit was computed to indicate if the misfit of the data was large enough to be problematic [33].

#### Instrument performance improvement

Rasch analysis can be used to identify overlapping items measuring similar aspects of the construct and/or items that do not fit the model well; termed *item redundancy*. Fit statistics (MnSq values) indicated whether an item might be redundant and considered for removal from the model [32]. Overlapping items were also identified using the Rasch person-item map as those occupying the same location on the map. To confirm item redundancy as identified using fit statistics and/or the person-item map, it was also necessary to assess whether the content of the item overlapped with any aspect of another item. If two or more items were similar in content, this might indicate redundancy. Following item removal, fit statistics and person-item maps confirmed whether model fit was improved.

#### Unidimensionality

In Rasch analysis, structural validity is determined by confirming the unidimensionality of the construct [30]. Winsteps uses a Principal Components Analysis (PCA) to create potential secondary dimensions (termed contrasts) based on the unexplained variance of the residuals, measured in eigenvalue units. The Winsteps PCA of residuals is not interpreted in the same way as Factor Analysis (FA) of the original data in classical test theory (CTT). For this analysis, the threshold for good evidence of unidimensionality was provided by an eigenvalue of less than 2.0 on the first contrast; (larger eigenvalues indicated the need for further investigation) [33]. Where eigenvalues exceeded 2.0, a CTT factor analysis of the original data (FA) was used to evaluate unidimensionality further. The Kaiser-Meyer-Olkin measure tested sampling adequacy, and Bartlett’s Test of Sphericity was used to detect the presence of multiple factors.

An important assumption of the RM is that there is no local response dependency. Local response dependency can occur when items are related to each other in a way that is outside the latent trait the outcome scale is measuring [35]. Local response dependency was evaluated through the calculation of Yens Q3 statistics. It is commonly recommended that these values do not exceed r = 0.7 [33]. Christensen et al. (2017) proposed that a single critical threshold for Q3 statistics was not appropriate for all situations and that a Q3 value of 0.2 above the average correlation was appropriate [35]. Local response dependency was assessed using both thresholds.

#### Differential item functioning (DIF)

DIF tested whether subgroups responded differently to items of the instrument compared with the rest of the sample. There is evidence of DIF when an item’s difficulty estimate location on the latent trait varies between subgroups by more than the modelled error [32]. There are two types of DIF. Uniform DIF provides information about whether the outcome scale performs similarly in subgroups while the item difficulties and person measures are held constant. Non-uniform DIF tests the performance of the outcome scale across subgroups at different levels of ability. To evaluate DIF Winsteps uses the Mantel Chi-Squared test with (log-)odds estimates of DIF size and tests significance from a comparison of the two groups. DIF that exceeds 0.64 logits is considered to be moderate to large [33]. The following demographic variables were used for DIF testing: gender, highest educational level (secondary vs tertiary) and signal joint (hip vs knee).

#### Non-Rasch tests of reliability and construct validity

Internal consistency was estimated to measure the level of interrelatedness between the items using Cronbach’s Alpha from CTT [36]. The threshold for Cronbach’s alpha was set at 0.8. The construct validity of the PAM-13 was explored using hypothesis testing [36]. Previous studies in different populations indicated PAM-13 scores were associated with the presence of depressive symptoms and health-related quality of life [37–39]; hence we expected moderate correlations between DASS and AQoL scores with PAM-13 (r > 0.3). We hypothesized that weak correlations (if any) would be observed between PAM-13 and HOOS/KOOS ‘Pain’ and ‘Function in daily living’ subscale scores (r < 0.2). Pearson’s correlations were used for normally distributed variables, Spearman’s correlations for those that were non-parametric. The thresholds for correlation size were defined as the following: ≥ 0.50 was large, 0.30–0.49 moderate, and 0.10–0.29 small [40].

## Results

### Study population

Out of the 238 participants consecutively enrolled in the OAMP February 2016 to June 2017 and approached to take part in the study, 21 participants declined to participate. The characteristics of the participants who completed the PAM-13 are summarised in Table 2. The group excluded based on non-completion was not large enough to make statistical comparisons.

**Table 2:** Characteristics of participants

Characteristics	Included *n* = 217	Excluded *n* = 21
**Sex** female, n (%)	148 (68)	10 (48)
**Age** years (SD)	65.5 (10.8)	68.7 (10.3)
**Signal joint** knee n (%)	183 (84)	15 (71)
**Body mass index** kg/m^2^(SD)	30.3 (6.1)	30.0 (5.1)
**PAM raw score**		
**PAM score** mean (SD)^a^	60.5 (11.0)	
PAM level 1 n (%)	10 (4.6)	
PAM level 2 n (%)	47 (21.7)	
PAM level 3 n (%)	123 (56.7)	
PAM level 4 n (%)	28 (12.9)	
**Highest level of education**		
Year 10 or equivalent n (%)	57 (26)	4 (19)
Year 12 or equivalent n (%)	28 (13)	1 (5)
Graduate degree n (%)	104 (48)	11 (52)
Post graduate degree n (%)	22 (10)	1 (5)
Missing n (%)	6 (3)	4 (19)
**Work status**		
Home duties n (%)	6 (3)	0
Full time n (%)	56 (26)	3 (14)
Part-Time n (%)	21 (10)	3 (14)
Retired n (%)	100 (46)	8 (38)
Volunteer n (%)	4 (2)	1 (5)
Other n (%)	25 (12)	2 (10)
Missing n (%)	5 (1)	4 (19)
**Private hospital** n (%)	160 (74)	21 (100)
**Public hospital** n (%)	57 (26)	0
**Average pain in last week on VAS**^b^	*n* = 214	n = 17
**VAS** mean (SD)	4.0 (2.3)	3.4 (2.5)
**KOOS**^c^	*n* = 179	*n* = 12
Pain mean (SD)	52.3 (17.7)	53.6 (14.1)
Function in daily living mean (SD)	58.0 (19.8)	59.3 (19.1)
**HOOS**^d^	*n* = 32	n = 3
Pain mean (SD)	57.7 (19.2)	61.7 (16.7)
Function in daily living mean (SD)	59.5 (18.1)	48.3 (20.2)
**DASS-21**^e^	n = 214	*n* = 16
Depression mean (SD)	7.2 (8.6)	5.2 (6.8)
Anxiety mean (SD)	5.1 (7.3)	3.8 (4.3)
Stress mean (SD)	8.8 (8.5)	7.5 (5.2)
**AQoL**^f^	*n* = 198	*n* = 6
Independent living mean (SD)	68.4 (19.3)	68.5 (15.5)
Social relationships mean (SD)	75.4 (19.8)	86.7 (10.3)
Mental health mean (SD)	69.8 (21.9)	74.0 (11.6)
Coping mean (SD)	65.3 (20.3)	69.3 (8.8)
Pain mean (SD)	45.8 (22.3)	55.0 (18.7)
Physical senses mean (SD)	81.8 (10.9)	79.7 (6.5)
AQoL summed index mean (SD)	68.4 (14.9)	72.1 (8.6)

^a^PAM: Patient Activation Measure- 0 = worst, 100 = best, however, participants with scores of 0 or 100 were excluded from having a final score. PAM level 1 = least activated, 4 = most activated

^b^ VAS: Visual analogue scale- average pain over the last week 0 = no pain, 10 = worst pain imaginable

^c^ KOOS: Knee injury and Osteoarthritis Outcome Score- 0 = worst, 100 = best

^d^ HOOS: Hip disability and Osteoarthritis Outcome Score- 0 = worst, 100 = best

^e^ DASS: Depression Anxiety Stress Scales- 0 = best, 42 = worst

^f^ Assessment of Quality of Life Instrument- Standardised scores- 0 = worst, 100 = best

### Item response frequency analysis

**Fig. 1:**
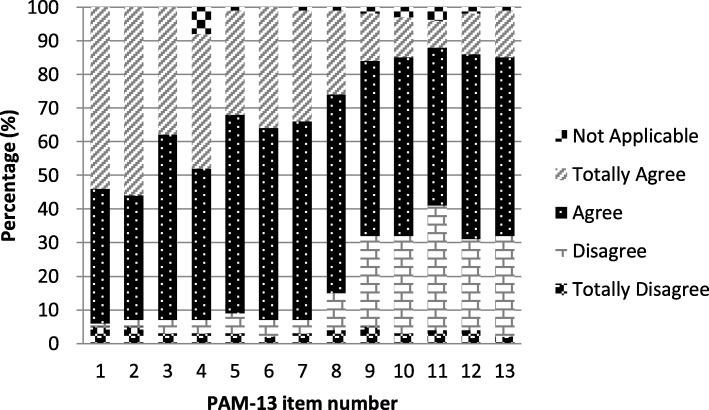
Responses across the PAM-13 agreement and not applicable categories (*n* = 217)

Of 217 attempted PAM-13 surveys, there were no missing responses, however, the N/A responses were not included in the scoring and were treated as missing data [41]. The distribution of responses to the questions is depicted in Fig. 1. The questions most commonly responded to with N/A were PAM-13 item-4 (I know what each of my prescribed medications do) and item-11 (I know how to prevent problems with my health), although N/A responses only comprised 2% (49/2821) of the total responses to these items. The most frequent response category overall was “agree” which comprised 1458/2821 (52%) of the total responses, followed by “totally agree” with 813/2921 (29%) responses. The “disagree” and “totally disagree” categories were much less frequent comprising 403/2821 (14%) and 98/2821 (3%) of all responses, respectively.

The mean response scores (range 1–4) for each item decreased from 3.4 (SD 0.73) for item-1 to 2.6 (SD 0.70) for item-11 (see Table 1). Although the mean response demonstrated an overall trend of decreasing as the questions became more difficult with subsequent items, the individual item order did not follow the originally established order of the questions [10]: for example, the mean for item-11 (mean 2.6, SD 0.70) was smaller than the means for item-12 and item-13 (mean 2.8, SD 0.70). Floor and ceiling effects were not detected; 1 % (2/217) and 3 % (7/217) of participants answered with ‘totally disagree” and “total agree” to all items respectively.

### Reliability and separation

The person and item reliability of the PAM-13 was adequate as indicated by; person reliability index 0.87 (> reference value 0.8), item reliability index 0.98 (> reference value 0.8). The person separation index was 2.58 (> reference value 2) indicating good separation.

### Rasch model fit analysis

**Fig. 2:**
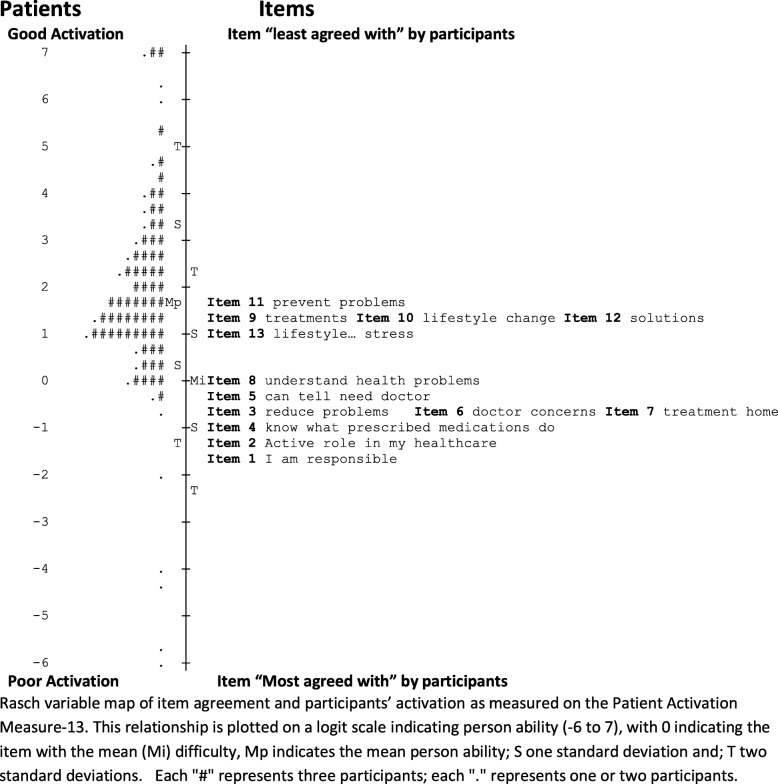
Person-item map of study participants and PAM-13 items

There were high positive point measure correlations of r = 0.58–0.78 for all PAM-13 items. The relationship between the mean difficulty of the items and the ability of participants expressed in logits is depicted in Fig. 2. Overall, the mean difficulty of the PAM-13 questions was lower than the mean ability of this sample. The mean PAM-13 item difficulty was shown at 0 logits, and the mean response of participants was almost 2 logits higher, 37% (81/217) people had abilities that exceeded the two most difficult items. Figure 2 also shows that the items were not evenly spread with several items having very similar item difficulty (see items 3,6 and 7; items 9, 10 and 12). Moreover, the item difficulty did not ascend uniformly with each subsequent item. This is confirmed by mean item difficulty calibrations (Table 3) which showed item difficulty order was different to the original PAM-13. The item difficulty of item-4 (I know what each of my prescribed medications do) was lower than item-3 (I am confident I can help prevent or reduce problems associated with my health. Item-5 (I am confident that I can tell whether I need to go to the doctor or whether I can take care of a health problem myself) had higher item difficulty than items-6 and -7 (6. I am confident that I can tell a doctor concerns I have even when he or she does not ask; 7. I am confident that I can follow through on medical treatments I may need to do at home). The greatest deviation between expected and actual order of difficulty was found for item-13 (I am confident that I can maintain lifestyle changes, like eating right and exercising, even during times of stress) which was lower than items 9–12.

**Table 3:** Item fit statistics for the PAM-13

PAM items (in order of difficulty)	Difficulty calibration (logits)	Infit Mean squared	Outfit Mean squared
1	−1.52	1.21	1.13
2	−1.50	**1.58**	**1.97**
4	−1.07	1.27	1.16
6	−0.75	0.80	0.75
3	−0.75	0.95	0.89
7	−0.55	0.95	1.04
5	−0.40	0.85	0.80
8	0.11	0.74	0.70
13	1.14	1.12	1.44
9	1.17	0.91	0.95
10	1.19	0.96	0.97
12	1.23	0.96	0.96
11	1.68	0.93	1.29

Note: Results in bold indicate values that are beyond the ideal cutoffs for infit and outfit statistics (i.e. Msq of 0.5–1.5)

Fit statistics are summarised in Table 3. Items fit the RM apart from item-2 with infit and outfit statistics of 1.58 and 1.97 Msq respectively, indicating under-fit. However, the global fit statistic indicated overall adequate fit of the data to the model (log-likelihood χ ^2^ = 3901.0644, 3927 + − 5 degrees of freedom, *P* = 0.612).

### Unidimensionality and structural validity

The Rasch dimension demonstrated that the persons and items within the analysis explained 49.4% of the variance (49.8% was expected if the sample fit model perfectly), with an eigenvalue of 12.70. The first contrast gave an eigenvalue of 2.5. Unidimensionality was further assessed using FA. The data was adequate for the FA (Kaiser-Meyer-Olkin value = 0.88 and Bartlett’s Test of Sphericity χ^2^ = 1404.0, df 78, *p* < 0.001). Using a scree plot and principal axis factoring, the PAM-13 loaded on one factor which explained 45.0% of the variance and suggested unidimensionality. In the assessment of local response dependence, the Yens Q3 values did not exceed the first common threshold of r = 0.7 suggesting the absence of local response-dependence. According to the second threshold, five items exceeded the Q3 value of 0.2 above the average correlation indicating the presence of local response dependence.

### Differential item functioning

No significant uniform DIF was found for people with hip OA compared with those with knee OA. There was significant uniform DIF for item-13 (I am confident that I can maintain lifestyle changes, like eating right and exercising, even during times of stress) which was more easily endorsed by women; (DIF contrast = 0.98 logits, Mantel chi-squared statistic χ^2^_M_ = 11.83_,_*p* = 0.001) compared to men. Item-7 (I am confident that I can follow through on medical treatments I may need to do at home) was easier to endorse for people who reported their highest educational level was tertiary vs those whose highest level was high school (DIF contrast =0.85 logits, χ^2^_M_ = 4.67, *p* = 0.031). Conversely, people whose highest level of education was high school found item-11 (I know how to prevent problems with my health) easier to endorse than those with tertiary level education (DIF contrast = 0.68 logits, χ^2^_M_ = 6.25, *p* = 0.012). The subgroups tested in this sample were not large enough to test for non-uniform DIF.

### Instrument performance improvement

**Fig. 3:**
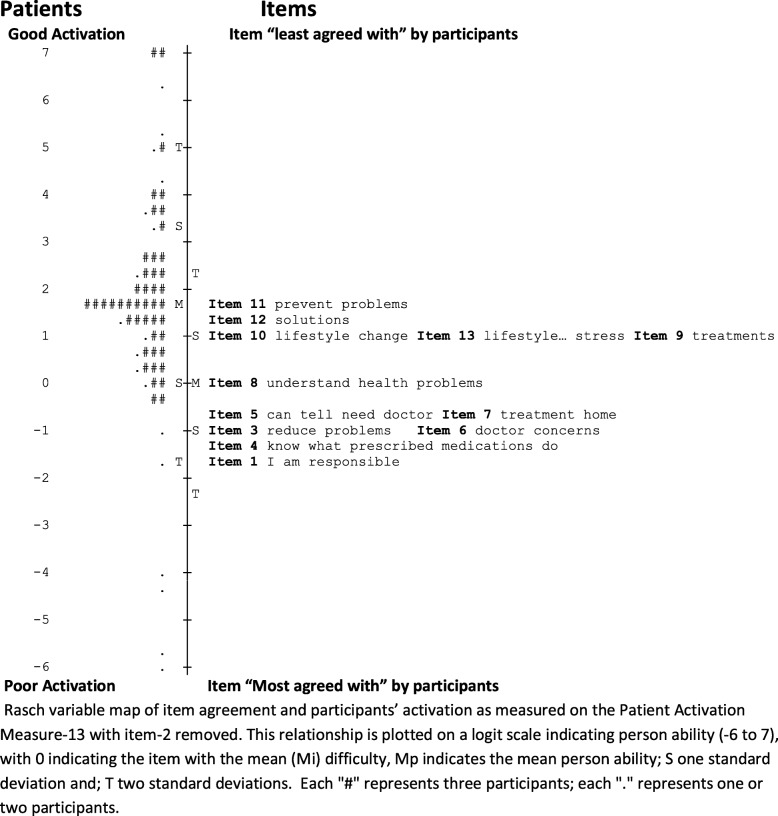
Person-item map of study participants and PAM-13 following removal of item-2

The person ability and item responses were assessed on person-item maps that depicted the logit values for all possible response options. The person-item map in Fig. 2 summarises the mean logit response across all response options. There were overlapping of items and similar item difficulties for item-3 (I am confident I can help prevent or reduce problems associated with my health), item-6 (I am confident that I can tell a doctor concerns I have even when he or she does not ask) and item-7 (I am confident that I can follow through on medical treatments I may need to do at home) seen in Fig. 2 and Table 3. It was decided that these items measured distinct aspects of the construct and they were deemed inappropriate for removal. Similarly, items 9, 10 and 12 were overlapping (Fig. 2), however, measured different aspects of the construct and were retained. Item-2 demonstrated poor fit statistics and was similar in item difficulty and content to item-1 (Table 3) so was removed. Removal of item-2 resulted in a slight improvement in the spread of the PAM-13 items (Fig. 3). Reliability remained adequate (person and item reliability 0.87 and 0.98, respectively) and there were high positive point measure correlations (r = 0.61 to r = 0.79). The fit statistics for the revised model (Table 4) revealed item-1 outfit statistic 1.56 MnSq, while the remaining items were acceptable. The PCA showed 49.9% of the variance was explained by the model (compared with 50.0% expected) with an eigenvalue of 12.0. The first contrast resulted in an eigenvalue of 2.2. The analysis following removal of item-2 did not improve the performance of the instrument adequately to recommend removal of this item in this population.

**Table 4:** Item fit statistics for the PAM-13 following removal of items − 2

PAM items (in order of difficulty)	Difficulty calibration	Infit Mean squared	Outfit Mean squared
1	−1.73	1.40	**1.56**
4	−1.25	1.32	1.19
6	−0.92	0.83	0.78
3	−0.92	1.04	0.99
7	−0.72	1.00	1.11
5	−0.55	0.88	0.84
8	−0.02	0.75	0.7
13	1.07	1.14	1.42
9	1.10	0.90	0.93
10	1.13	0.95	0.95
12	1.17	0.97	0.97
11	1.65	0.92	1.23

Note: Results in bold indicate values that are beyond the ideal cutoffs for infit and outfit statistics (ie. Msq of 0.5–1.5)

### Non-Rasch tests of reliability and construct validity

The estimated Cronbach’s Alpha indicated adequate internal consistency α = 0.92 (> reference value 0.8). The correlations between the PAM-13 scores and other variables are summarised in Table 5. Lower activation scores were moderately correlated with the presence of depressive symptoms on the DASS; r = − 0.26, (95% Confidence interval (CI) -0.38, − 0.14). Higher activation scores correlated moderately with higher health-related quality of life score as measured on the AQoL; r = 0.32 (95% CI 0.18, 0.47). There were small correlations between PAM-13 and KOOS pain and ADL scores (r = 0.13 (95% CI 0.03, 0.29) and r = 0.15 (95% CI 0.03, 0.31) respectively). There were no significant correlations between PAM-13 and HOOS pain or function scores.

**Table 5:** Correlations between Patient Activation Measure-13 scores and other variables

	Expected correlation^a^	Actual correlation	95% Confidence interval
DASS depression^s^ (*n* = 205)	<−0.3	− 0.26	− 0.38, − 0.14
AQoL (*n* = 190)	> 0.5	0.32	0.18, 0.47
KOOS Pain (*n* = 171)	< 0.2	0.13	0.03, 0.29
KOOS ADL (n = 171)	< 0.2	0.15	0.03, 0.31
HOOS Pain^a^ (*n* = 31)	< 0.2	−0.06	− 0.47, 0.39
HOOS ADL^a^ (n = 31)	< 0.2	−0.23	− 0.54, 0.15

Pearson’s correlations were used for normally distributed variables, ^a^Spearman’s correlations for those that were non-parametric. DASS: Depression Anxiety Stress Scales- 0 = best, 42 = worst. AQoL: Assessment of Quality of Life Instrument- Standardised scores- 0 = worst, 100 = best. KOOS: Knee injury and Osteoarthritis Outcome Score- 0 = worst, 100 = best. HOOS: Hip disability and Osteoarthritis Outcome Score- 0 = worst, 100 = best.

## Discussion

### Discussion

Adequate person and item reliability was demonstrated for the PAM-13 and unidimensionality was evaluated. There were some issues with targeting items to people with higher abilities and the item-order was different from that expected for the PAM-13. Rasch analysis revealed that item-2 under-fit the model and its removal resulted in a very slightly improved model fit, but not enough to recommend its removal. There was evidence of a difference in item response based on sex and educational status, though this was limited to a small number of items. The presence of depressive symptoms and AQoL scores correlated moderately with PAM-13 as expected.

International studies commonly report a different item difficulty order to the original order for PAM-13 published in American cohorts [10, 12, 15, 42–44]. This was consistent with the findings of our study with the exception of three items: − 1 (When all is said and done, I am the person who is responsible for taking care of my health); − 2 (Taking an active role in my own health care is the most important thing that affects my health) and − 12 (I am confident I can figure out solutions when new problems arise with my health) [10]. These were also the only items consistent with the original order in a Canadian study of participants with neurological conditions [12]. Items-1 and -2 alone followed the original order of item difficulty in a study of adults in Korea living with OA [15]. Item-1 was the ‘easiest’ item in a Danish study [43], but not in studies of the German and Italian PAM-13 in people with chronic conditions [42, 44]. Differences in item-difficulty order seen in our study and other populations may be attributed to specific disease and cultural factors. The differences in self-management tasks required and corresponding difficulty of these should be considered in the context of the health conditions and populations in which the PAM-13 is used.

The unidimensionality of the PAM-13 was assessed; almost 50% of the variance was explained by the items and participant responses. This percentage of explained variation was higher than reported in other disease populations [42–44], but not as high as that reported for the Korean version of PAM-13 tested in an OA sample (57.5%) [15]. The limited proportion of variance explained suggests there may be other additional factors that comprise this construct that are not captured by the items of the instrument. On the other hand, it may indicate simply that the items were of similar difficulty and the participants in the study were of similar ability [33]. This study relied on the PCA of Rasch residuals and conventional factor analysis. Further information on the unidimensionality of PAM-13 in this population using other statistical tests such as confirmatory factor analysis based on polychoric correlations or further Rasch based tests may be valuable in future research.

The assessment of local response dependence using two different thresholds of Q3 values yielded conflicting results. The conventional threshold Q3 value indicated the absence of local response dependence. The second threshold of the mean Q3 + 0.2 suggested the presence of local response dependence. To confirm these results, it would be helpful to attempt to replicate these results in future studies. This could also provide further evidence regarding the potential for different results produced by commonly recommended thresholds versus thresholds that are influenced by the characteristics of the dataset being analysed.

There were issues identified with targeting of the PAM-13 items. Looking at the person-item map from this study (Fig. 2), the ability of the participants most often exceeded the difficulty of the items. The lack of items of sufficient difficulty could affect the precision of the measure for people with greater ability (i.e. greater probability of agreeing or partially agreeing with the items). A possible way of dealing with both the low proportion of explained variance and limited targeting of items to people with higher abilities may be to develop an OA-specific version of the PAM-13 in a similar way the version was developed for mental health (PAM-MH) [45]. An important implication of modifying the PAM-13 to be condition-specific would be the loss of the ability to compare populations and the relative impact of different medical conditions and/or treatments. Further, people with OA commonly report the presence of several chronic comorbidities [46]; it is arguably more useful to use a generic instrument and consider self-management of health in general.

There were a few incidences of significant uniform DIF in this study., Significant DIF was found for item-13 (I am confident that I can maintain lifestyle changes, like eating right and exercising, even during times of stress) suggesting that women find it more difficult to endorse this item. A systematic review synthesised determinants of adherence to lifestyle intervention in adults with obesity [47] and found that being female was a predictor of attrition from lifestyle interventions. Given that the mean BMI for our study sample was 30.3 m/kg^2^, most of the participants were overweight/obese, so this could offer some evidence relating to why women found it more difficult to endorse this item. This finding is consistent with one study of PAM-13 in a different population (Italian speaking people with chronic diseases) [42], however, there was no significant DIF identified for gender in the OA Korean PAM-13 study [15].

Our analysis found that PAM Item-7 (I am confident that I can follow through on medical treatments I may need to do at home) was easier to endorse for those people with higher formal educational level. This result conflicted with the findings of an Italian study reporting that people with higher education levels found this item more difficult to endorse [42]. There is existing evidence that suggests people with higher education levels feel more confident in self-management of their OA. A large cohort study found that people with OA, who had higher educational levels, reported higher Arthritis Self Efficacy Scale (ASES) scores. The ASES measures the ability of people to manage the symptoms of their OA [48]. Interestingly, another finding in our study was that item-11 (I know how to prevent problems with my health) was harder to endorse for participants with higher education levels. This is an unexpected finding, we would expect people with higher educational levels to be confident of not only how to manage their OA at home, but also of how to prevent problems. This result was not reported in other studies of the PAM-13. The DIF identified for three PAM-13 items in this study indicates potential bias in the measurement of patient activation in subgroups of people living with hip and knee OA. This should be investigated in future studies and if DIF is found for the same items, there are several ways that this could be managed, such as removal of those items [49].

This is the first time, to our knowledge that a study, has examined measurement properties of the English language version PAM in a sample of people living with OA. There is growing interest in the utility of the PAM-13, particularly in the United Kingdom where the PAM-13 is being appraised as a tool used to evaluate care for chronic conditions in the National Health Service [50]. It is important to improve our understanding of the measurement properties of PAM-13 in different disease populations and this study is a valuable contribution to this growing body of evidence.

There are some limitations to the applicability of this study which included a fairly homogenous population from a higher socio-demographic region of Australia. Future studies should aim to include a less geographically and socio-demographically homogenous sample. There was also a large proportion of people in our study with knee OA so that the sample was less representative of people with hip OA. Future studies should assess larger groups of participants with hip OA to ensure that accurate measurement properties are available for people with this disease.

## Conclusions

There is limited extant measurement property evidence available to support the use of any instrument assessing OA self-management attitudes and capabilities. This study provides evidence of adequate person and item reliability, unidimensionality, and construct validity to support the use of PAM-13 to measure patient activation in people living with OA. Potential areas for concern regarding the PAM-13 responses from this sample include possible local response dependence, DIF and issues with targeting. Further studies of the measurement properties of the PAM-13 in people with OA are recommended for the purposes of research, and to provide information about how the PAM-13 can be used with individual OA patients in the clinic.

## Data Availability

The datasets used and/or analysed during the current study are available from the corresponding author on reasonable request.

## Abbreviations

PAM-13: Patient Activation Measure 13 item version
OA: Osteoarthritis
DIF: Differential item functioning
OAMP: Osteoarthritis management program
HREC: Human Research Ethics Committee
N/A: Not applicable
HOOS: The hip disability and osteoarthritis outcome score
KOOS: Knee injury and Osteoarthritis Outcome Score
DASS-21: Depression, Anxiety and Stress Scale 21 item version
AQoL-6D: Assessment of Quality of Life 6 dimension version
BMI: Body mass index
RM: Rasch model
CTT: Classical test theory
MnSq: Mean square
PCA: Principal components analysis
FA: Factor analysis
SD: Standard deviation
χ^2^: Chi squared
df: Degrees of freedom
χ^2^_MH_: Mantel-Haenszel chi-squared statistic
PAM-MH: Patient Activation Measure Mental Health version
